# Years of Experience and Its Association with Indicators of Adiposity and Health-Related Quality of Life in Teachers: A Cross-Sectional Study

**DOI:** 10.3390/healthcare14121694

**Published:** 2026-06-13

**Authors:** Andrés Godoy-Cumillaf, Josivaldo de Souza-Lima, Maribel Parra-Saldias, Daniel Duclos-Bastias, Claudio Farias-Valenzuela, Eugenio Merellano-Navarro, José Bruneau-Chávez

**Affiliations:** 1 Grupo de Investigación en Educación Física, Salud y Calidad de Vida (EFISAL), Facultad de Educación, Universidad Autónoma de Chile, Temuco 4780000, Chile; 2 Facultad de Educación y Humanidades, Escuela de Ciencias del Deporte, Universidad Andres Bello, Las Condes, Santiago 7550000, Chile; josivaldo.desouza@unab.cl; 3 Departamento de Educación Física, Deporte y Recreación, Universidad de Atacama, Copiapó 1530000, Chile; maribel.parra@uda.cl; 4 iGEO, Escuela de Educación Física, Facultad de Filosofía y Educación, Pontificia Universidad Católica de Valparaíso, Valparaíso 2340025, Chile; daniel.duclos@puvc.cl; 5 METIS Research Lab, Facultad de Negocios y Tecnología, Universidad Alfonso X el Sabio (UAX), 28691 Madrid, Spain; 6 Escuela de Ciencias de la Actividad Física, el Deporte y la Salud, Universidad de Santiago de Chile (USACH), Santiago 9170022, Chile; claudio.farias.v@usach.cl; 7 Department of Physical Activity Sciences, Faculty of Education Sciences, Universidad Católica del Maule, Talca 3530000, Chile; emerellano@ucm.cl; 8 Departamento de Educación Física, Deportes y Recreación, Universidad de la Frontera, Temuco 4811230, Chile; jose.bruneau@ufrontera.cl

**Keywords:** BMI, WHRT, WHR, HRQoL

## Abstract

**Background/Objectives**: Teachers in educational institutions are continuously exposed to high occupational demands, which may contribute to the development of increased adiposity and comparatively unfavorable health-related quality of life (HRQoL) scores. However, there is limited evidence regarding how years of professional experience is associated with these indicators in teachers. The objective of this study is to examine the association between years of professional experience, adiposity indicators, and HRQoL among teachers in educational institutions. **Methods**: An observational, relational, exploratory cross-sectional study was conducted in 175 teachers from educational institutions in the city of Temuco, Chile. Body mass index (BMI), waist-to-height ratio (WHtR), and waist-to-hip ratio (WHR) were assessed as adiposity indicators, and health-related quality of life (HRQoL) was measured using the SF-12. Age, sex, and years of professional experience were recorded. Simple and multivariable linear regression models were used to analyze the association between years of experience and the study variables, adjusting for age and sex. Additionally, experience tertiles were compared using ANOVA and ANCOVA. **Results**: In the unadjusted analyses, greater years of professional experience were associated with higher adiposity indicators, including BMI (β = 0.071; 95% CI: 0.020 to 0.129). However, after adjustment for age and sex, these associations were attenuated and no longer statistically significant (adjusted BMI: β = −0.172; 95% CI: −0.434 to 0.053). Associations with PCS and MCS scores were also not statistically significant after adjustment. **Conclusions**: Teachers exhibited high levels of adiposity and HRQoL scores suggesting an unfavorable perceived health profile. The observed associations between years of professional experience and adiposity or HRQoL appear to be largely explained by age rather than by professional experience itself. Future longitudinal studies are needed to more precisely distinguish between the effects of aging and prolonged occupational exposure. However, the findings should be interpreted cautiously given the convenience sampling design and the inclusion of teachers from a single city.

## 1. Introduction

In recent years, evidence in occupational health has indicated that work organization and the work environment are associated with physical and mental health outcomes, including fatigue, stress and reduced perceived well-being [1,2]. In this context, teachers working in educational establishments, who perform an essential role in the functioning of educational systems, work under conditions that directly influence their health. They are frequently exposed to a combination of sustained demands, such as high workload, pressure to achieve results and emotional demands, which may lead to the adoption of unhealthy behaviors, particularly in contexts where time for self-care is limited [2,3,4,5].

Within this framework, studies conducted with teachers in educational establishments have shown that they present high levels of adiposity, indicating a health profile that warrants attention [6,7]. This is particularly relevant, as elevated adiposity constitutes a health risk factor [8] and is associated with an increased risk of myocardial infarction and cardiometabolic complications [9,10].

Another affected aspect in teachers is health-related quality of life (HRQoL), defined as the individual’s perception of how their health status influences their daily life [11]. Evidence on HRQoL in teachers indicates that it is negatively affected by health conditions and the occupational characteristics of their work environment [12,13].

Given that the work performed by teachers in educational establishments exposes them to high workload demands on a sustained basis [2,3,4,5], teaching can be conceptualized as an occupation involving chronic exposure, which may have adverse effects on health in both the short and long term. In this context, years of professional experience may represent an occupationally meaningful indicator of cumulative exposure to the teaching environment, including prolonged psychosocial demands, sedentary behavior, workload intensity, and work-related stressors accumulated throughout the professional trajectory [2,3,4,5,14,15]. However, because years of professional experience is strongly linked to chronological age, distinguishing the specific effects of accumulated occupational exposure from broader age-related processes represents an important methodological challenge in cross-sectional research. Therefore, examining years of professional experience in relation to adiposity and HRQoL may provide exploratory insight into how occupational trajectory and aging-related processes overlap in teachers’ health profiles. Research in occupational health suggests that current work models are associated with greater adiposity and changes in body weight, supporting the existence of a relationship between psychosocial working conditions and adiposity [14,15]. This is further supported by evidence indicating that greater excess weight is associated with poorer HRQoL, while weight reduction is generally accompanied by improvements in HRQoL [16], underscoring the importance of studying adiposity alongside quality of life.

However, although teachers have been described as presenting high levels of adiposity and lower HRQoL [6,7,12,13], there is limited information on whether years of work experience is associated with adiposity indicators and HRQoL, which would allow for the identification of potentially more vulnerable stages and provide evidence to support the development of health promotion and prevention strategies. Given that evidence on the relationship between work experience, adiposity and quality of life in teachers remains limited, the present study aimed to evaluate these associations in this specific population as an initial, exploratory step towards future comparative research across occupations. Therefore, the objective of this study was to examine the association between years of work experience, adiposity indicators and health-related quality of life in teachers working in educational establishments.

## 2. Materials and Methods

### 2.1. Design and Participants

An observational, relational, exploratory cross-sectional study with a quantitative approach was conducted. A convenience sampling strategy was used, inviting primary and secondary school teachers working in educational establishments in the city of Temuco, La Araucanía region, Chile, to participate. The invitation was carried out via email and direct contact with the teachers. Given the exploratory nature of the study and the use of convenience sampling, no a priori sample size calculation was performed.

**Figure 1:**
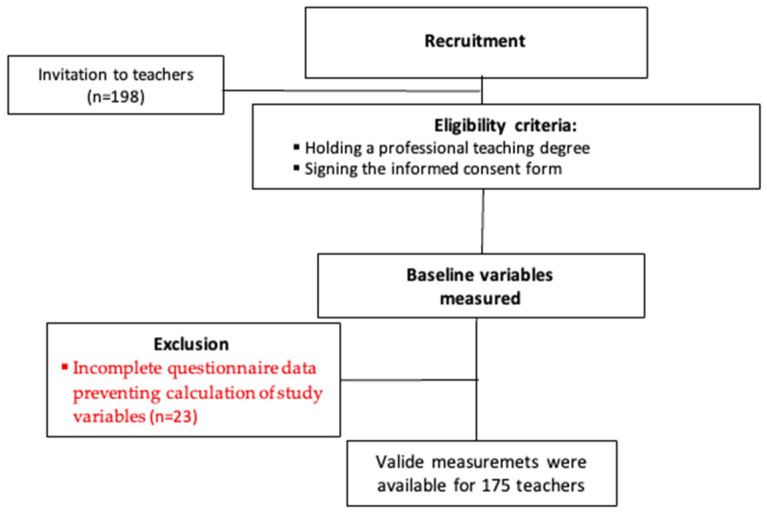
Participant recruitment and exclusion flowchart.

The inclusion criteria were holding a professional teaching degree and signed the informed consent form. The initial sample consisted of 198 teachers. After completion of data collection and data cleaning, the final analytical sample comprised 175 teachers (Figure 1). Participants with incomplete questionnaire data that prevented calculation of the study variables were excluded from the final analyses.

The study protocol was approved by the Scientific Ethics Committee of Universidad Autónoma de Chile (No. 22-25). All procedures were conducted in accordance with the principles of the Declaration of Helsinki.

### 2.2. Instruments

Body mass index (BMI) was calculated by dividing body weight (kg) by height (m) squared. Body weight was assessed using a digital scale (Omron), with participants wearing minimal clothing and no footwear. Height was measured using a stadiometer (Seca Model 200, seca GmbH & Co. KG, Hamburg, Germany), with participants barefoot, standing upright and looking straight ahead. The measurement was taken at the end of a normal inspiration. Both measurements were taken in duplicate, and the mean value of each was used for BMI calculation.

Waist circumference was measured using a tape measure (Holway, Thousand Oaks, CA, USA), with the participant standing in an upright position. On both sides of the trunk, the last rib and the upper border of the iliac crest were located, and the midpoint between these anatomical landmarks was identified as the site for tape measure placement. Hip circumference was also measured using a tape measure (Holway), with the participant in the same position as described for waist circumference measurement. This value was obtained at the most prominent part of the buttocks, at the level of the pubic symphysis. Both measurements were taken in duplicate, and the mean value of each was used to calculate the waist-to-height ratio (WHtR) and the waist-to-hip ratio (WHR). WHtR was determined by dividing waist circumference (cm) by height (cm), while WHR was determined by dividing waist circumference (cm) by hip circumference (cm). BMI, WHtR and WHR have been described as reliable indicators of adiposity [17,18,19,20].

Health-related quality of life was assessed using the SF-12 questionnaire [21], which comprises 12 items scored on a Likert-type scale. Through eight dimensions (physical functioning, physical role, bodily pain, general health, vitality, mental health, social functioning and emotional role), the questionnaire yields quality of life scores grouped into two summary components: the Physical Component Summary (PCS) and the Mental Component Summary (MCS). PCS and MCS scores were calculated following the standard scoring procedures described by Ware et al. [21], using the original SF-12 scoring algorithm derived from general US population norms. Scores for each component range from 0 to 100, with higher scores indicating better health-related quality of life. Scores were analyzed as continuous indicators of perceived health status, and no missing SF-12 items were observed in the analyzed sample.

Age, sex and years of work experience were collected through a self-report form completed by each participant prior to the evaluations. Years of teaching work experience was operationalized as the number of years elapsed since obtaining the professional teaching degree. This variable was used as an exploratory proxy indicator of accumulated occupational exposure in the teaching profession. However, it does not capture actual years actively worked in teaching, potential interruptions in the work trajectory, delayed entry into professional practice, differences in workload intensity, teaching hours, administrative responsibilities, or variability in psychosocial working conditions over time.

### 2.3. Procedure

Data were collected between September and December 2025. Evaluations were scheduled with each teacher via email, instant messaging or telephone call, and were always conducted between 8:00 and 10:00 a.m. On the scheduled date, a member of the research team traveled to the educational establishment where the teacher or teachers to be evaluated were working.

Each evaluation began with the signing of the informed consent form and the completion of the self-report form to collect data on age, sex and years of work experience. This was followed by the administration of the health-related quality of life questionnaire. Subsequently, body weight, height, waist circumference and hip circumference were measured. The entire process lasted approximately 10 to 12 min per participant. Data collection was carried out by graduates in physical education, who were previously trained to ensure standardization in the anthropometric measurements and to address any questions arising from the questionnaire. Although formal inter-rater reliability and technical error of measurement were not quantified, all evaluators received prior standardized training following the same anthropometric assessment procedures.

### 2.4. Statistical Analysis

Normality of the variables was assessed using the Kolmogorov–Smirnov test. Sex differences were examined using the independent samples Student’s *t*-test. To evaluate the association between years of work experience and the study variables (BMI, WHtR, WHR, PCS and MCS), simple linear regression models were fitted.

Additionally, to address the potential confounding effect of age, multivariable linear regression models were fitted, including using years of work experience as the main predictor and adjusting for age and sex. Given the expected collinearity between chronological age and years of professional experience, adjusted analyses were interpreted cautiously and were considered exploratory rather than confirmatory with respect to potential occupational-exposure effects. Additionally, multicollinearity diagnostics were assessed using variance inflation factor (VIF) and tolerance statistics. The adjusted models were intended to partially account for basic demographic confounding and should not be interpreted as causal models. The assumptions of linearity (residuals versus fitted values plots), normality of residuals (Q–Q plots and Shapiro–Wilk test) and homoscedasticity (visual inspection of residuals) were assessed. Results are reported as unstandardised coefficients (β) with 95% confidence intervals.

As an exploratory complementary analysis, years of work experience was also categorized into tertiles. Differences between tertiles were evaluated using one-way ANOVA. Furthermore, ANCOVA was conducted to compare tertiles while adjusting for potential confounders, including age and sex. Bonferroni correction was applied in post hoc comparisons.

A *p*-value < 0.05 was considered statistically significant. Statistical analyses were performed using SPSS software, version 29 (IBM Corp., Armonk, NY, USA).

## 3. Results

The characteristics of the sample, for the whole group and stratified by sex, are presented in Table 1. Statistically significant differences between men and women were observed for body weight, height, BMI, waist circumference, WHR and MCS.

Chronological age and years of professional experience were strongly positively correlated (r = 0.945, *p* < 0.001), indicating substantial overlap between both variables. Consistent with this finding, multicollinearity diagnostics showed elevated VIF values for chronological age (VIF = 9.40) and years of professional experience (VIF = 9.40), with low tolerance values (0.106), indicating substantial multicollinearity between both variables.

Table 2 presents the results of the association between years of work experience and the study variables, examined through simple linear regression (Model 1) and multivariable linear regression in which years of work experience was the main predictor, adjusted for age and sex (Model 2).

In the unadjusted models, years of work experience was positively associated with adiposity indicators. Specifically, each additional year of work experience was associated with higher BMI (β = 0.071; 95% CI: 0.020 to 0.129), WHtR (β = 0.003; 95% CI: 0.001 to 0.004), and WHR (β = 0.003; 95% CI: 0.001 to 0.005). In contrast, the associations with HRQoL were less clear, as the 95% confidence intervals for both PCS (β = −0.079; 95% CI: −0.174 to 0.020) and MCS (β = 0.018; 95% CI: −0.064 to 0.101) included zero.

After adjustment for age and sex, the evidence for an independent association between years of work experience and the study outcomes was no longer clear. The adjusted estimates were β = −0.172 for BMI (95% CI: −0.434 to 0.053), β = 0.000 for WHtR (95% CI: −0.004 to 0.004), β = 0.002 for WHR (95% CI: −0.002 to 0.007), β = −0.248 for PCS (95% CI: −0.604 to 0.061), and β = 0.154 for MCS (95% CI: −0.120 to 0.398). In all adjusted models, the 95% confidence intervals included zero. Taken together, these results indicate that years of work experience was associated with adiposity indicators only in the unadjusted analyses; after accounting for age and sex, these associations were not maintained, suggesting that the unadjusted pattern largely reflects the overlap between chronological age and years of work experience.

Complementary analyses based on tertiles of work experience showed patterns consistent with the regression analyses, with crude differences attenuating after adjustment for age and sex. Detailed results are presented in Supplementary Table S1.

## 4. Discussion

The present study explored the association between years of work experience, adiposity indicators and health-related quality of life (HRQoL) in Chilean teachers working in educational establishments. Overall, the results show that teachers presented adiposity indicators that warrant attention from an occupational health perspective and comparatively unfavorable HRQoL scores, suggesting an overall health profile of concern within this occupational group. These findings regarding adiposity are consistent with evidence linking central obesity with metabolic disturbances and adverse health outcomes [22,23,24].

Recent literature has shown that the distribution of adiposity and central obesity are associated with hormonal dysfunction and cardiometabolic risk in both men and women, and that relevant sex differences exist in the accumulation of adipose tissue [22,23]. In this context, the use of indicators such as BMI and WHtR is particularly appropriate, given that the latter has been proposed as a simple and useful tool for predicting mortality and cardiometabolic risk across different populations [24]. Our results, showing unfavorable values for these indicators among teachers, reinforce the need to consider adiposity as a central component in the characterization of the health profile of this occupational group.

Furthermore, evidence in occupational health has indicated that factors such as daily sedentary time, repeated exposure to adverse psychosocial work factors and long working hours are associated with greater cardiometabolic risk and long-term deterioration of cardiovascular health [25,26,27,28]. Recent narrative evidence has also emphasized the important role of physical activity in occupational well-being, stress reduction, productivity, and overall health outcomes in working populations [28]. Sedentary behavior has been associated with an unfavorable cardiometabolic profile in older adults [25], while repeated exposure to psychosocial risk factors at work and long working hours in middle age have been linked to increased arterial stiffness and a poorer cardiovascular profile in later stages of life [26,27]. Within this framework, it is plausible to consider that the combination of sustained work demands and sedentary lifestyles may contribute to the accumulation of health risk in teachers throughout their work trajectory.

Regarding HRQoL, recent studies in teachers have shown that various health- and behavior-related factors, such as tobacco use and non-compliance with 24 h movement guidelines, are associated with poorer quality of life and a greater burden of physical and mental symptoms [29,30,31]. It has been reported, for example, that tobacco use in teachers is bidirectionally associated with poorer quality of life [29]; that adherence to recommendations on physical activity, sleep and sedentary behavior is linked to better quality of life in school teachers [30]; and that musculoskeletal disorders in Chilean teachers are associated with significant deterioration in quality of life [31]. Our results, which suggest an unfavorable perceived health profile in this population, are consistent with this body of evidence and underscore that the health of teachers is affected by the interaction of multiple occupational, behavioral and physical health factors. Importantly, the associations between years of work experience and HRQoL were weaker and less consistent than those observed for adiposity indicators, and they were substantially attenuated after adjustment for age and sex. This suggests that HRQoL in teachers is likely influenced by a complex interaction of psychosocial, behavioral, occupational, and health-related factors that extend beyond professional seniority alone. Therefore, the present findings reinforce the importance of adopting multidimensional approaches when evaluating well-being and perceived health in teaching populations.

A specific contribution of this study is the explicit analysis of the relationship between years of work experience and adiposity indicators and HRQoL, while accounting for its close relationship with chronological age. The added value of examining years of work experience separately from age lies in the fact that work experience represents an occupationally meaningful indicator of accumulated exposure to the teaching context, whereas chronological age captures broader biological, behavioral, and life-course processes. Although this indicator is imperfect and may not fully capture interruptions in professional activity or differences in occupational intensity over time, it may still reflect aspects of chronic occupational exposure not entirely represented by chronological age alone. Evaluating both variables therefore allowed us to assess whether professional seniority provided information beyond known age-related effects. In the unadjusted models, a greater number of years of work experience was associated with higher adiposity values and poorer physical HRQoL. However, upon incorporating age and sex into the multivariable models, the magnitude of these associations was substantially reduced and, in some cases, no longer reached statistical significance. This pattern is informative because it suggests that professional seniority, although relevant from an occupational health perspective, did not show a clear independent association with adiposity or HRQoL beyond chronological age in this sample. Furthermore, the adjusted models explained only a modest proportion of the variance in the study outcomes, and the attenuation of the regression coefficients after adjustment suggests that years of professional experience contributed little independent explanatory value beyond chronological age and sex. Importantly, the very high correlation and elevated VIF values observed between chronological age and years of professional experience indicate substantial multicollinearity, which may also have reduced the statistical precision and power of the adjusted models to detect independent associations. Therefore, one plausible interpretation of the present findings is that older teachers, rather than teachers with longer occupational exposure per se, tend to present worse adiposity indicators.

An important implication of these findings is that years of work experience should not be interpreted as an independent determinant of adiposity or HRQoL in this sample. Because work experience is closely intertwined with chronological age, the observed associations likely reflect a combination of aging-related and occupational processes that cannot be clearly disentangled in this cross-sectional design. Accordingly, the findings should be interpreted as exploratory statistical associations rather than evidence of causal relationships.

These findings are further situated within a context in which other factors relevant to teachers’ health, such as sedentary behavior, physical activity, sleep, tobacco use and the presence of musculoskeletal disorders, have been shown to be associated with quality of life and health status in this population [25,29,30,31]. This suggests that the relationship between years of work experience, adiposity and HRQoL is likely mediated or modified by a set of behavioral and occupational factors that were not directly measured in the present study, and which may account for part of the differences observed among teachers with varying work trajectories.

This study has several limitations that should be considered when interpreting the results. First, the cross-sectional design precludes the establishment of directionality in the associations between years of work experience, adiposity and HRQoL. In particular, a key limitation for interpretation is the difficulty in disentangling the effects of years of work experience from those of chronological age. Although both variables were considered in the analyses, they are inherently closely related, and in a cross-sectional design, their independent contributions cannot be clearly separated. Additionally, no a priori sample size calculation was performed due to the exploratory nature of the study and the use of convenience sampling. Although the final sample included 175 teachers, the very strong correlation between chronological age and years of work experience may have reduced the statistical power to detect an independent association of work experience after adjustment for age. Therefore, non-significant adjusted associations should be interpreted cautiously and should not be considered definitive evidence of absence of association. Consequently, the associations initially observed with work experience should be interpreted cautiously, as they may primarily reflect age-related biological, behavioral, and life-course processes rather than a specific influence of accumulated teaching exposure. Therefore, the findings should be understood as statistical associations rather than causal relationships. Additionally, the use of convenience sampling and the inclusion of teachers from a single city may limit the external validity and generalizability of the findings to other teacher populations or educational contexts. Moreover, teachers who agreed to participate may have differed systematically from those who declined participation, potentially introducing selection bias related to health status, lifestyle behaviors, or occupational characteristics. Second, although the models were adjusted for age and sex, information on other potential confounding factors was not available, including physical activity, dietary patterns, working hours, sleep quality and the presence of comorbidities, all of which have been associated with both cardiometabolic risk and HRQoL [24,25,26,27,30]. The absence of these variables increases the likelihood of residual confounding. For example, teachers with longer professional trajectories may differ from younger teachers in lifestyle behaviors, occupational demands, or chronic disease burden, which could partially explain the observed associations with adiposity and HRQoL. Differences in physical activity, sedentary behavior, sleep quality, dietary patterns, and cardiometabolic comorbidities may therefore have contributed to the attenuation or persistence of some associations after adjustment. Therefore, the adjusted associations reported in this study should not be interpreted as approximations of causal effects, but rather as exploratory estimates within the limitations of the available data. Additionally, because the study did not include a comparison population or nationally representative normative data, the interpretation of HRQoL scores should be considered descriptive rather than diagnostic. Third, years of work experience were operationalized as the number of years elapsed since obtaining the professional teaching degree, which represents an approximation of effective work experience and does not accurately capture delayed entry into the profession, interruptions in the work trajectory, or periods of employment outside the school setting, potentially introducing non-differential misclassification of the exposure. Therefore, this variable should be interpreted as an indirect and exploratory proxy of occupational exposure rather than a precise measure of cumulative teaching exposure. Future longitudinal studies should prioritize repeated assessments of physical activity, sedentary behavior, dietary intake, sleep quality, workload characteristics, psychosocial occupational stressors, and chronic disease status to better distinguish occupational exposure effects from aging-related processes.

Despite these limitations, the study has several strengths. It focuses on a professional group that plays a key role in society and has been identified as vulnerable from a health perspective, within a Latin American context that remains underrepresented in the literature [29,30,31]. Furthermore, it simultaneously examines multiple adiposity indicators and both dimensions of HRQoL, providing a broader picture of teachers’ health status than that offered by studies relying on a single indicator [22,23,24]. Importantly, by explicitly addressing the collinearity between age and years of work experience, and by demonstrating how adjustment for age modifies the pattern of associations, the study contributes to a more nuanced understanding of how occupational seniority should be interpreted in epidemiological analyses and in the planning of preventive interventions in occupational health [24,25,26,27,29,30,31]. More broadly, these findings illustrate the methodological challenges of disentangling occupational seniority from life-course aging processes in cross-sectional occupational health research. Rather than simply demonstrating the expected correlation between age and occupational seniority, the present study illustrates how strongly intertwined these variables are in practice and how adjustment for chronological age substantially modifies the interpretation of associations commonly attributed to professional experience. In this sense, the study contributes methodological insight into the interpretation of occupational trajectory variables in cross-sectional occupational health research.

In summary, this exploratory study shows that Chilean teachers working in educational establishments present high levels of adiposity and comparatively unfavorable HRQoL scores, and that the apparent associations between years of work experience and these variables are largely explained by chronological age when both variables are considered simultaneously. These findings reinforce the need for occupational health promotion and prevention strategies targeting the teaching workforce and indicate that future research should employ longitudinal designs and more precise measurements of work trajectory and lifestyle factors to more clearly distinguish between age-related processes and the specific impact of prolonged occupational exposure [22,23,24,25,26,27,29,30,31].

## 5. Conclusions

This exploratory study provided evidence that Chilean teachers working in educational establishments presented adiposity indicators and HRQoL scores suggestive of an unfavorable health status, reinforcing the importance of preventive and occupational health strategies targeting this population. In the unadjusted analyses, a greater number of years of work experience was associated with higher adiposity indicators, whereas the associations with HRQoL were less clear. After adjustment for age and sex, however, the 95% confidence intervals for all outcomes included zero, indicating that years of work experience did not show a clear independent association with adiposity indicators or HRQoL beyond chronological age and sex. Therefore, these findings should be interpreted cautiously. In this dataset, years of work experience were closely entangled with chronological age and may largely reflect age-related biological, behavioral, and life-course processes rather than the specific effect of accumulated occupational exposure. Accordingly, the present design does not allow for a robust distinction between the effects of aging and accumulated occupational exposure.

The main contribution of these exploratory findings is therefore to underline the importance of considering age and work trajectory simultaneously when assessing teachers’ health, as well as the need to develop occupational health promotion and prevention strategies targeting this group, particularly in contexts characterized by high work demands and limited opportunities for self-care. Future studies, ideally with longitudinal designs and a more detailed characterization of work trajectory, lifestyle factors and occupational exposure will be essential to more precisely distinguish between age-related processes and the specific impact of prolonged occupational exposure.

## Figures and Tables

**Table 1:** Sample characteristics.

	Total (Mean ± SD) (*n* = 175)	Men (*n* = 58)	Women (*n* = 117)	*p*-Value
Age	39.59 ± 11.8	40.22 ± 12.44	39.28 ± 11.52	0.63
Weight (kg)	73.55 ± 13.96	82.74 ± 12.99	69 ± 12.1	**0.01**
Height (cm)	163.78 ± 7.00	170.67 ± 5.00	160.36 ± 5.00	**0.02**
BMI (kg/m^2^)	27.37 ± 4.64	28.44 ± 4.6	26.84 ± 4.58	**0.03**
Waist (cm)	87.3 ± 16.04	94.16 ± 18.08	83.91 ± 13.78	**0.00**
Hip (cm)	100.67 ± 9.63	100.16 ± 10.07	100.93 ± 9.44	0.62
WHTR	0.53 ± 0.09	0.55 ± 0.1	0.52 ± 0.09	0.08
WHR	0.87 ± 0.13	0.94 ± 0.14	0.83 ± 0.11	**0.00**
Years of experience	13.85 ± 11.35	14.41 ± 11.78	13.56 ± 11.18	0.64
PCS	49.52 ± 7.29	49.52 ± 7.29	48.25 ± 7.07	0.21
MCS	41.39 ± 3.90	43.39 ± 3.90	41.23 ± 4.84	**0.00**

The values in bold indicate a statistical significance of *p* < 0.05.

**Table 2:** Association between years of work experience, adiposity indicators and health-related quality of life.

	β Model 1	CI 95%	β Model 2	CI 95%
BMI (kg/m^2^)	0.071	0.020 to 0.129	−0.172	−0.434 to 0.053
WHtR (RCE)	0.003	0.001 to 0.004	−0.000	−0.004 to 0.004
WHR (ICC)	0.003	0.001 to 0.005	0.002	−0.002 to 0.007
PCS (SF-12)	−0.079	−0.174 to 0.020	−0.248	−0.604 to 0.061
MCS (SF-12)	0.018	−0.064 to 0.101	0.154	−0.120 to 0.398

Model 1: unadjusted; model 2: adjusted by age and sex. BMI: body mass index; WHtR: waist-to-height ratio; WHR: waist-to-hip ratio; PCS: physical component summary; MCS: mental component summary; CI: confidence interval.

## Data Availability

The data presented in this study are available on request from the corresponding author. The data are not publicly available due to ethical restrictions.

## Supplementary Materials

The following supporting information can be downloaded at: https://www.mdpi.com/article/10.3390/healthcare14121694/s1, Table S1: Comparison of study variables across tertiles of work experience.

## Abbreviations

The following abbreviations are used in this manuscript:

WHTR	waist-to-height ratio
WHR	waist-to-hip ratio
BMI	Body mass index
HRQoL	health-related quality of life
