# Supplementary material for: Years of Experience and Its Association with Indicators of Adiposity and Health-Related Quality of Life in Teachers: A Cross-Sectional Study
Source: Healthcare (Basel). 2026 Jun 13;14(12):1694. doi: 10.3390/healthcare14121694 (PMC13299429; doi:10.3390/healthcare14121694)
Supplement: Supplementary file 1 [file healthcare-14-01694-s001.zip › healthcare-4202395-supplementary.pdf]

In the ANOVA analysis, significant overall differences between tertiles were observed for the adiposity indicators BMI (p = 0.002), WHR (p = <0.001) and WHtR (p = <0.001). In contrast, no significant differences between tertiles were identified for PCS (p = 0.210) or MCS (p = 0.870).

After adjusting for age and sex (ANCOVA), differences between tertiles were attenuated and no longer statistically significant for BMI (p = 0.080), WHR (p = 0.340) and WHtR (p = 0.820), nor for PCS (p = 0.790) or MCS (p = 0.160). In the adjusted model, no significant pairwise differences between tertiles were observed after correction for multiple comparisons, further supporting the interpretation that the apparent differences in the unadjusted analysis are largely explained by age differences between groups.

Table S1. Comparison of study variables across tertiles of work experience.

	Tertile 1(n=59) (1 to 5 years)	Tertile 2 (n=62) (6 to 18 years)	Tertile 3 (n=54) (19 to 43 years)	p-value ANOVA	p-value ANCOVA
BMI	26.03±3.40	28.08±5.59^{T3}	28.02±4.35^{T2}	**0.002**	0.080
WHTR	0.49±0.06^{T3}	0.54±0.10	0.56±0.09^{T1}	**<0.001**	0.820
WHR	0.83±0.09^{T3}	0.86±0.15	0.9±0.12^{T1}	**<0.001**	0.340
PCS	51.29±6.29	49.61±6.73	49.03±8.33	0.210	0.790
MCS	50.25±5.14	49.68±6.81	50.08±6.47	0.870	0.160

Values are presented as mean ± standard deviation. Bold values indicate statistical significance (p < 0.05). Superscripts (T1, T2, T3) indicate statistically significant differences compared with the corresponding tertile in post hoc comparisons (Bonferroni correction, p < 0.05).
